# Construction, Evaluation, and Optimization of a Regional Ecological Security Pattern Based on MSPA–Circuit Theory Approach

**DOI:** 10.3390/ijerph192316184

**Published:** 2022-12-03

**Authors:** Chunguang Hu, Zhiyong Wang, Gaoliu Huang, Yichen Ding

**Affiliations:** 1 School of Architecture and Urban Planning, Huazhong University of Science and Technology, Wuhan 430074, China; 2 Hubei Engineering and Technology Research Center of Urbanization, Wuhan 430074, China

**Keywords:** morphological spatial pattern analysis (MSPA), circuit theory, ecological security pattern (ESP), Taihu Lake Basin, construction–evaluation–optimization

## Abstract

Ecological security is crucial for regional sustainable development; however, as modern urbanization highlights ecological security challenges, major challenges have arisen. In this paper, we take the ecological region around Taihu Lake, China, as a typical research site, extract important ecological sources and key nodes using morphological spatial pattern analysis (MSPA) and circuit theory, and propose a regulatory framework for the ecological security pattern (ESP) of the ecological region based on the spatial characteristics of sources, corridors, and nodes. We obtained the following results: (1) The ESP includes 20 ecological sources, 37 ecological corridors, 36 critical ecological protection nodes, and 24 key ecological restoration nodes. (2) Most ecological sources are large and concentrated in western Zhejiang and west of Taihu Lake, which are both important ecological sources and ecological resistance surfaces. (3) The ecological corridors spread east, west, and south from Taihu Lake, with high network connectivity. (4) Shanghai serves as the central node, with the Su-Xi-Chang town cluster and the Qiantang River town cluster serving as the extension axes for the ecological resistance hot-spot area. The center of the elliptical ecological resistance surface (standard deviation) lies in Suzhou City, located on the east shore of Taihu Lake. (5) Ecological nodes were mostly located in ecological corridors or junctions. A “four zones and one belt” pattern is suggested in order to make the land around Taihu Lake more connected and stable ecologically. This study can be used as a guide for building and improving an ecological safety network.

## 1. Introduction

When it comes to ensuring sustainable regional development, ecological safety is the starting point and a necessary pre-condition. The loss of habitat, fragmentation, and other threats to biodiversity have resulted in a steady decline in both the number of animals and the quality of the regional ecological environment [1,2,3]. On the other hand, ecosystems are fragile and sensitive, so the intensification of human activities will inevitably cause damage to the ecological security pattern (ESP) [1,4]. The rise of urbanization, in particular, has a negative impact on the ESP, increasing the level of unpredictability that regional eco-systems must deal with [5]. For this reason, more and more countries are realizing how important the ESP is and incorporating it into their national security systems [6,7]. Researching how to ensure regional ESP is also a key part of regional sustainable development [5,8].

The effective identification of the ESP is an important method for the rational planning of ecological regions [9]. First, the ESP typically entails building ecological sources, resistant surfaces, ecological corridors, and ecological pinch points [10]. As a result, by employing the “point–line–plane” approach, the ESP can make it easier for creatures to move between habitats while maintaining the integrity of the ecosystem structure. This can protect biodiversity against the long-term effects of habitat fragmentation [11]. Among these are ecological sources, which are the patches that play an important role in biodiversity conservation. In the past, in the process of source selection, scholars have generally used qualitative ecosystem types [12], quantitative habitat importance [13], ecological sensitivity [14], and ecosystem services [3] to unify the sources in the ESP. The preceding considerations are adequate for determining the commonality of sources but are insufficient for studying their characteristics. For example, the ecological corridors between recreation and nature reserve sources may not be suitable for either species migration or human recreation. Morphological spatial pattern analysis (MSPA), which focuses on structural connections [15], makes it easier to choose ecological sources in a scientific way. It also helps to define how species move through the landscape in an objective manner [16].

Currently, various research theories and methods have been developed for the construction of the ESP, including a graphical approach [17], circuit theory [18], and the Minimum Cumulative Model (MCR) [19]; the latter of which is based on the “source–sink” theory, and has become a mainstream assessment method due to its flexible additivity [5]. By computing resistance surfaces and subsequently extracting important ecological nodes and corridors, it has been widely utilized to pinpoint ecological sources and mimic ecosystem processes [20]. However, although this method can characterize the orientation of ecological corridors, it usually ignores the stochastic wandering behavior of species and fails to specify the specific extent (corridor width) and key nodes (pinch points) of corridors. As a result, in this work, we use circuit theory to forecast the motion patterns in complex landscapes, addressing a major limitation of the MCR model previously used to identify key landscape patches [21]. In summary, this paper combines the MSPA-Circuit theory to analyze regional ESP because the MSPA method places an emphasis on the system’s internal and structural connectivity, whereas circuit theory is employed to effectively make up for the MSPA method’s lack of functional connectivity and to locate crucial pathways in ecological systems [22,23]. By combining the two methods, conservation strategies for the ESP that are more scientifically sound can be developed.

It is noteworthy that most studies on the ESP have focused on the identification of ecological sources and the identification of research paradigms for ecological corridor construction but have typically neglected to effectively evaluate the ESP and provide subsequent optimization measures [5,8]. In the earlier days, the ESP construction was aimed at biodiversity conservation; however, with the development of an ecosystem service assessment and the recognition of the importance of ecological security [3,24], it was found that the ESP would then have an impact on local socio-economics [25,26], and the subsequent attention was gradually focused on the spatial structure of ecosystems [27], ecological functions and processes [28], coupled ecosystem services [29], and other related components [5]. Therefore, this study enhances the analysis process of evaluation and optimization for key elements that make up the ESP, such as patches, corridors, and nodes, in order to achieve the important goal of identifying effective management of the ESP at a regional scale.

At the same time, contemporary researchers have concentrated on the administrative boundaries set by prefecture-level cities [30], provinces [20], and urban agglomerations [31], as these boundaries are clear and relevant data are easily accessible, which does add some convenience to the study. However, the spatial development of a biological species can hardly be limited by artificial administrative boundaries. Therefore, in this paper, we attempt to propose a set of ecological safety planning schemes. For the region to grow in a healthy and sustainable way, administrative borders need to be broken down and a set of cross-regional plans for ecological security need to be made. The ecological region around Taihu Lake in China was selected as a typical and representative case site. The Yangtze River’s ecological regulator, China’s Taihu Lake Ecological Region, includes many administrative regions of Jiangsu and Zhejiang, has a shoreline of about 400 km, and is rich in ecological resources. Therefore, we need to build the ESP across regions as soon as possible to protect ecosystems and keep them working.

We employed the MSPA–Circuit theory to investigate the ESP of the ecological region around the Taihu Lake Basin, considering the following three major objectives: (1) to creatively propose a framework for the “construct–evaluate–optimize” research method for the ESP; (2) to explore the ESP characteristics under ecologically dominant functions; and (3) to propose a planning scheme for ecological safety protection and restoration for ecological regions. These goals are intended to provide strong theoretical support for cross-regional ecological security management.

## 2. Study Area

**Figure 1:**
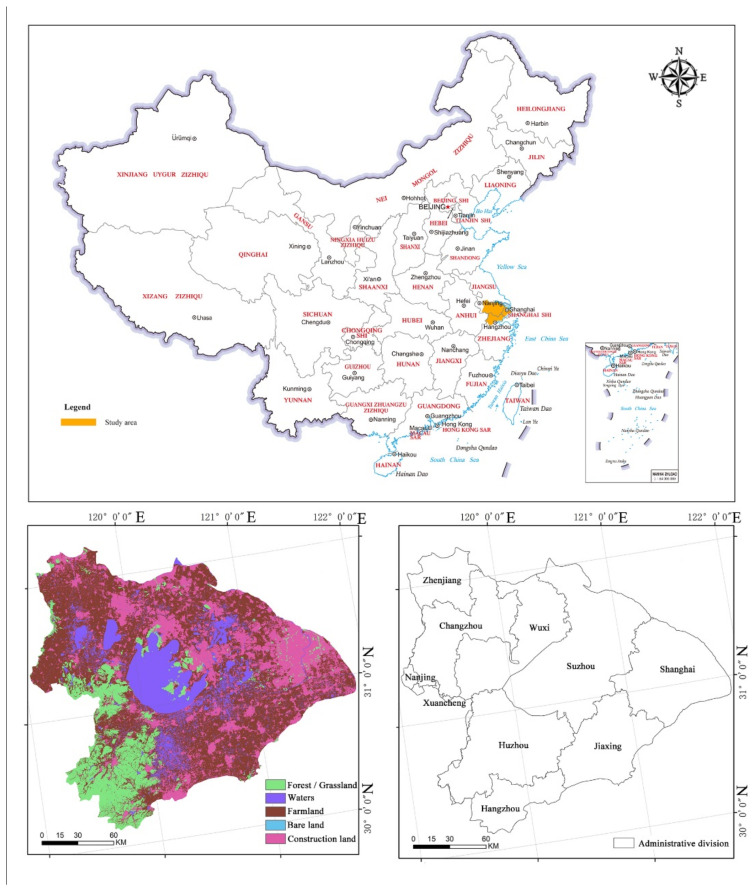
Location and distribution of different forms of land-cover around the Taihu Lake Basin (2020).

Taihu Lake Ecological Region is in the core area of the Yangtze River Delta in China (Figure 1). With a watershed area of 36,895 km^2^, it accounts for 0.4% of the country’s area while generating about one-eighth of the country’s GNP, making it a typical transboundary ecological protection and development area. On one hand, the watershed is home to megacities (represented by Shanghai), large and medium-sized cities (represented by Hangzhou and Suzhou), and numerous small cities and established towns that are rapidly developing. On the other hand, there are many lakes and dense river networks in the basin, with important beaches and wetlands serving as habitats for various migratory birds. This means that the biological environment and biodiversity of the region will eventually be stressed due to the processes of urbanization and development. Ecological security has, therefore, become a big problem for the region.

## 3. Research Framework and Data Sources

### 3.1. Research Framework

**Figure 2:**
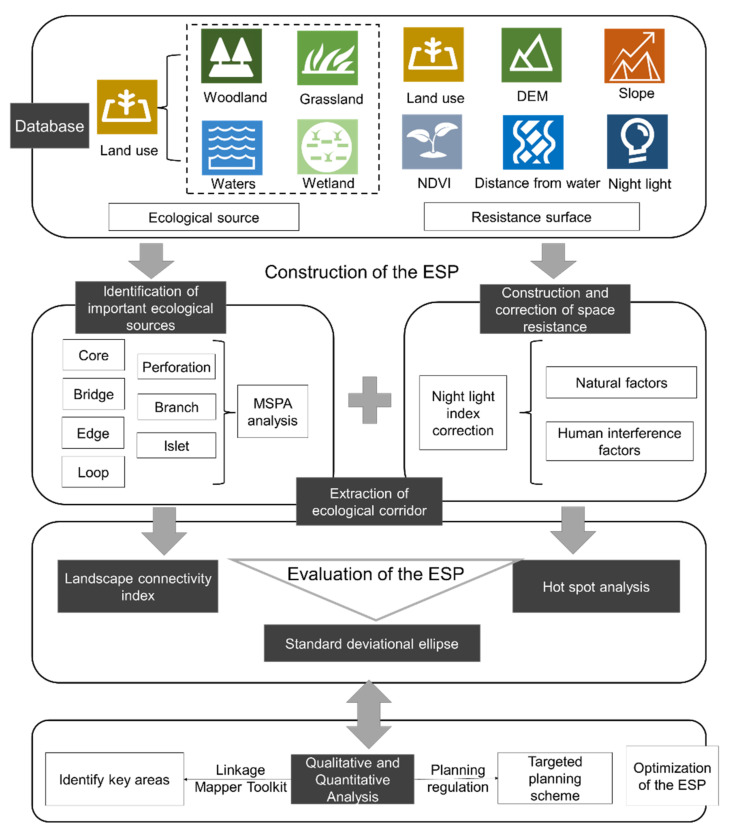
Research framework.

At present, the main study paradigm for developing the ESP includes determining the source area, constructing the resistance surface, and setting the corridor width. The three logical parts of the ESP are construction, evaluation, and optimization. After that, we made the research framework in Figure 2, which has the four steps below:

(1) Establishing a database—We focus on putting together a basic database for the study of the ESP based on data from both naturalistic and humanistic fields.
(2) Constructing the ESP—The MSPA method, emphasizing structural connectivity, is used to identify ecological sources. Natural factors affecting biological activity and spatiality are selected using weights obtained through AHP hierarchical analysis. Then, the anthropogenic disturbance factors are used as corrected spatial resistance factors. Ecological corridors are identified using Linkage Mapper, resulting in the ESP that links important ecological sources.
(3) Evaluating the ESP—The importance of ecological sources is assessed according to the connectivity index. Hot-spot analysis and the standard ellipse approach are used to characterize the spatiality of ecological resistance affecting the direction of species activity.
(4) Optimizing the ESP—We use both quantitative and qualitative approaches to optimize the ESP of the ecological region around Taihu Lake. The quantitative approach focuses on the spatial location of microscopic-specific locations, while the qualitative approach focuses on guidance and control at a larger scale.

First, we use the circuit theory method to find ecological key areas, such as ecological pinch points and barrier points, in order to determine ecological protection areas and ecological restoration areas in the study area. Then, we make sure we come up with the right measures for the protection and restoration areas that we have found, such that internal flaws and outside threats can be avoided. Second, we suggest targeted ESP optimization tactics and zoning control measures based on planning and management strategies at the district and county levels.

### 3.2. Data Sources

The data in this study mostly fall into three main categories: (1) the cross-administrative scope of the study area; (2) the basic data of ecological sources, based on the MSPA method, to study the ecological region around Taihu Lake; and (3) the multi-source natural–social basic data needed to calculate the integrated ecological resistance surface of the ecological region around Taihu Lake, as shown in Table 1. All the data were simultaneously combined into one coordinate system (WGS 1984, UTM Zone 50N) with the assistance of the ArcGIS 10.5 software, and re-sampled to a spatial resolution of 30 m × 30 m.

## 4. Methods

### 4.1. Identification of Important Ecological Sources

Combined with the current biodiversity situation in the ecological region around Taihu Lake, we consider the activity range and space of species represented by egrets. We take natural landscapes with high ecological service values and less human interference in woodlands, grasslands, and waters (including wetlands) as prospective data for the MSPA analysis. Arable land and construction land are not suitable as species activity spaces due to high human influence, and bare land lacks a living environment for species to forage. The above three land types were used as background data. Using the Guidos Toolbox analysis and a series of calculations on the data, seven landscape types were determined in the ecological region around Taihu Lake (Table 2).

The Conefor software (http://www.Conefor.org/, accessed on 16 May 2022) was used to compute the Probability of Connectivity (PC) and the incremental PC (DPC), which reflect the connectivity of patches to the landscape [33]. Egrets can move up to 10 km [34], and the patch connectivity distance was calculated using the following formulas:

(1) $$PC=\frac{\sum_{i=1}^{n}\sum_{j=1}^{n}a_{i}a_{j}p_{ij}^{*}}{A_{L}^{2}},$$

(2) $$dI\left(dPC\right)=\frac{I-I^{\prime }}{I}\times 100,$$

where *n* is the number of ecological patches, $a_{i}$ and $a_{j}$ are the areas of patches i and j, respectively, AL is the total area of the regional landscape, $p_{ij}^{*}$ is the maximum probability value of species diffusion between patches i and j, dI indicates the degree of importance of the removed elements ,I is the connectivity calculation result, and $I^{\prime }\;$indicates the connectivity calculation result after removing a particular element. If the dI value is higher, it indicates that the degree of patch connection is higher [35].

### 4.2. Construction and Correction of Space Resistance

The scientific construction of ecological resistance factors is important for the conservation of biodiversity in ecological sites. In terms of natural factors, the type of landscape influences whether it is suitable for the survival of the species. The activity range of a species is influenced by elevation and slope, and its density is influenced by the amount of vegetation present. The biological activity increases as one gets closer to a river. Therefore, elevation, slope, type of landscape, amount of vegetation, and distance from a river were chosen as natural resistance factor indicators in this work, based on previous literature research [36,37,38] and the real conditions around the Taihu Lake Basin (Table 3). The resistance values were put into groups, based on a previous field study of the Taihu Lake Basin, and the AHP was used to determine the importance of each factor.

**Figure 3:**
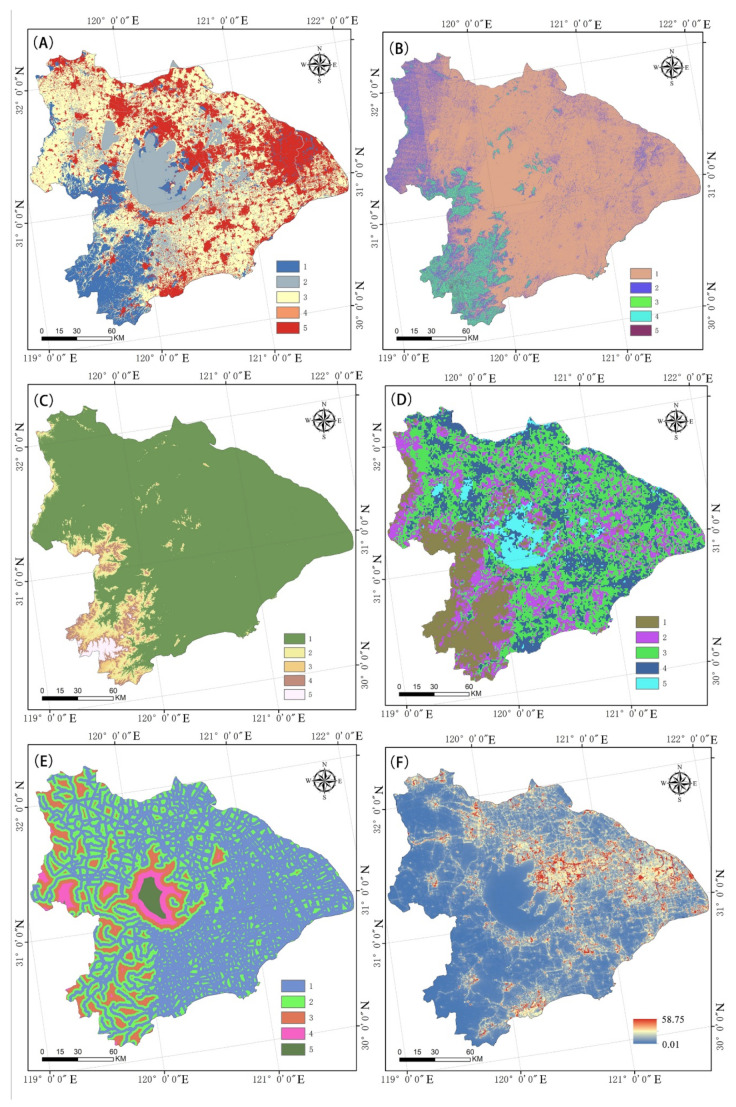
Various resistance factors around Taihu Lake Basin: (**A**) Land-use type resistance; (**B**) slope resistance; (**C**) elevation resistance; (**D**) NDVI resistance; (**E**) distance resistance to water; and (**F**) night light index.

As a result, it was split into five levels, numbered 1 through 5. The greater the value, the greater the ecological resistance to the spread of biological species. Figure 3 displays the ecological resistance factor requirements. Figure 3A depicts the re-classified land-use classification, Figure 3B shows the re-classified slope classification, Figure 3C shows the re-classified elevation classification, Figure 3D shows the re-classified NDVI classification, and Figure 3E shows the re-classified distance to water classification.

The primary determinants of the survival range of a species are natural forces, yet human actions have an impact on species that cannot be disregarded [39]. The influence of man-made disturbances is somewhat greater than that of natural forces [40], particularly in the Taihu Lake River basin, where the economy is growing quickly. As a result, the ecological resistance coefficient was modified using the night-time lighting index, which can be used to describe the level of human disturbance (Figure 3F). The equation is as follows:

(3) $$R^{*}=\frac{TLI_{i}}{TLI_{a}}\times R_{o},$$

where $R^{*}$ is the ecological resistance coefficient of the grid based on the night light index, $TLI_{i}$ is the night light intensity value of grid *I*, $TLI_{a}$ is the average light index of different resistance grades corresponding to grid *a*, and$\;R_{o}$ is the basic resistance coefficient of the raster landscape type.

### 4.3. Extraction of Ecological Corridor

The ArcGIS 10.5 software used in the study was licensed by ArcInfo, Link-age Mapper (http://www.circuitscape.org/lin-kagemapper, accessed on 18 May 2022) and was developed in ArcGIS to assess the regional connectivity of habitats for animals and plants [41]. The Linkage Pathways Tool is one of the tools in this toolkit, which can provide the required environmental data to use tools, such as Euclidean distance, which allow for the determination of ecological corridors and connections/bridges between ecological sources [42].

### 4.4. Spatial Analysis of Ecological Resistance

Only by understanding the issues faced by various regions can we construct a pattern of ecological security more pertinently [2], thereby elucidating the viability of building a corridor network around the Taihu Lake tourist area and the scientific nature of the optimization of the ESP, combined with the spatial characteristics of ecological resistance.

First, a fishing net measuring 1000 m in length was constructed in the study region, using the ecological resistance as a base. The geographical traits of the ecological resilience in the basin were determined using the Getis-Ord G* method, often known as hot-spot analysis. This was calculated as follows [3]:

(4) $$G_{i}^{*}=\frac{\sum_{j=1}^{n}w_{i,j}x_{j}-\bar{X}\sum_{j=1}^{n}w_{i,j}}{\frac{S\sqrt{\left[n\sum_{j=1}^{n}w_{i,j}^{2}-{\left(\sum_{j=1}^{n}w_{i,j}\right)}^{2}\right]}}{\left(n-1\right)}},$$

(5) $$\bar{X}=\frac{1}{n}\sum_{j=1}^{n}x_{i},$$

(6) $$S=\sqrt{\frac{1}{n}\sum_{j=1}^{n}x_{j}^{2}-{\left(\bar{X}\right)}^{2}},$$

where $x_{j}$ represents the amount of ecological resistance change of spatial unit *j*, $w_{i,j}$ represents the binary spatial weight matrix, *n* represents the number of spatial units, and the higher the Z-score of the $G_{i}^{*}$ index, the tighter the aggregation of hot-spots; on the other hand, the lower the score, the tighter the aggregation of cold-spots.

Secondly, the ecological resistance surface values at 1000 randomly chosen points within the research region were determined. The standard deviational ellipse (SDE) approach was used to further investigate the directional properties of ecological resistance. From a global and spatial point of view, the following equation can be used to explain how ecological resistance is centralized, distributed, and shaped in space [43]:

(7) $$x^{\prime }=x_{i}-x_{\mathrm{ave}},y^{\prime }=y_{i}-y_{\mathrm{ave}},$$

(8) $$\tan\theta =\left(\left(\sum_{i=1}^{n}W_{i}^{2}x_{i}^{\prime 2}-\sum_{i=1}^{n}W_{i}^{2}y_{i}^{\prime 2}\right)+\sqrt{\left(\sum_{i=1}^{n}W_{i}^{2}x_{i}^{\prime }y_{i}^{\prime }-\sum_{i=1}^{n}W_{i}^{2}y_{i}^{\prime 2}\right)+4\left(\sum_{i=1}^{n}W_{i}^{2}x_{i}^{\prime }y_{i}^{\prime }\right)}\right)\times {\left(2\sum_{i=1}^{n}W_{i}^{2}x_{i}^{2}y_{i}^{2}\right)}^{-1},$$

(9) $$\delta_{x}=\sqrt{\frac{\sum_{i=1}^{n}{\left(W_{i}x_{i}^{\prime }\cos\theta -W_{i}y_{i}^{\prime }\sin\theta \right)}^{2}}{\sum_{i=1}^{n}W_{i}^{2}}},$$

(10) $$\delta_{y}=\sqrt{\frac{\sum_{i=1}^{n}{\left(W_{i}x_{i}^{\prime }\sin\theta -W_{i}y_{i}^{\prime }\cos\theta \right)}^{2}}{\sum_{i=1}^{n}W_{i}^{2}}},$$

where $\left(x_{\mathrm{ave}},y_{\mathrm{ave}}\right)$ is the average center of$\;\left(x_{i},y_{i}\right)$, $W_{i}$ is the ecological resistance, and $\left(x^{\prime },y^{\prime }\right)$ are the relative coordinates of each point from the center of gravity of the study area. The azimuth angle can be obtained from tanθ, and $\delta_{x}$ and $\delta_{y}$ and are the standard deviations of the *x*-axis (short axis) and *y*-axis (long axis), respectively.

### 4.5. Identify Key Areas

#### 4.5.1. Identify Key Ecological Protection Nodes

Ecological conservation nodes are key components of species migration and energy flow in an ecoregion [44]. We utilized the Pinchpoint Mapper—a plug-in that is part of the Linkage Mapper toolkit—to identify important environmentally protected nodes [45]. The base map of Linkage Mapper was connected to the Circuitscape method through the Pinchpoint Mapper [46]. In order to supplement the results regarding the lowest-cost corridor, the maps were constructed to show pinch locations in that corridor, as well as useful resistance values for Linkage Mapper [47]. Pinchpoint Mapper finds places where the loss of a small area could make the landscape less connected in a significant manner [48,49].

#### 4.5.2. Identify Key Ecological Restoration Nodes

Key ecological restoration nodes serve as critical areas in an ecoregion where the flow of ecological elements is impeded. As a result, these locations have a significant impact on the connectivity of local ecological patches. The ecological environment and targeted ecological protection can be enhanced by integrating the ecological patch into the ecological restoration node. We utilized the Barrier Mapper plug-in, a component of the Linkage Mapper toolset, in order to locate essential ecological restoration nodes in corridors with the lowest cost [50]. More information on Linkage Mapper can be found in the associated program description [51,52,53].

## 5. Results

### 5.1. Construction of the ESP

**Figure 4:**
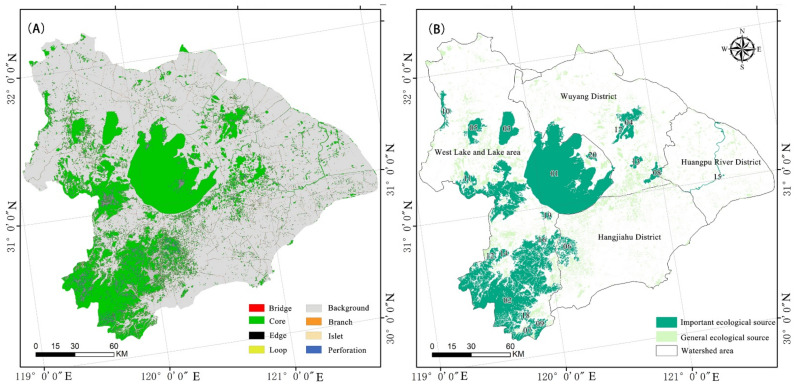
Important ecological sources around Taihu Lake Basin: (**A**) MSPA results; and (**B**) important ecological sources.

The results of MSPA-based ecological source identification in the ecological region around Taihu Lake are shown in Table 4 and Figure 4. The study area contained 27,186 regional core landscapes with a total area of 9145.86 km^2^, accounting for 85.23% of the total area of the extracted prospects. The marginal area adjacent to the research area’s core landscape was 1071.49 km^2^, which made up 9.99% of the entire foreground area. This indicates that the study area’s prospects had a great marginal effect. The connecting branch line and pore area accounted for 1.77% and 1.53% of the total potential area, respectively. The bridge region could not adequately facilitate the ecological flow of energy and matter, as it made up only 0.86% of the total foreground area. Furthermore, only 0.38% of the potential space was taken up by small islets, and only 0.24% of the total foreground area was found to support the spread of species through the ecological patches.

The core region comprised a sizable habitat patch, which could support a variety of creatures and contribute to biodiversity preservation. The first 20 core areas were chosen as viable ecological sources for the habitats of specific species, based on the size of the source area [7]. Taihu Lake, GE Lake, Yangcheng Lake, Chang-dang Lake, Chenghu Lake, Dianshan Lake, Huangpu River, and Xiazhu Lake were some of the source locations. The biological source areas in the forest and grasslands were generally separated by the rugged and hilly area southwest of the Taihu Lake tourist region. 

**Figure 5:**
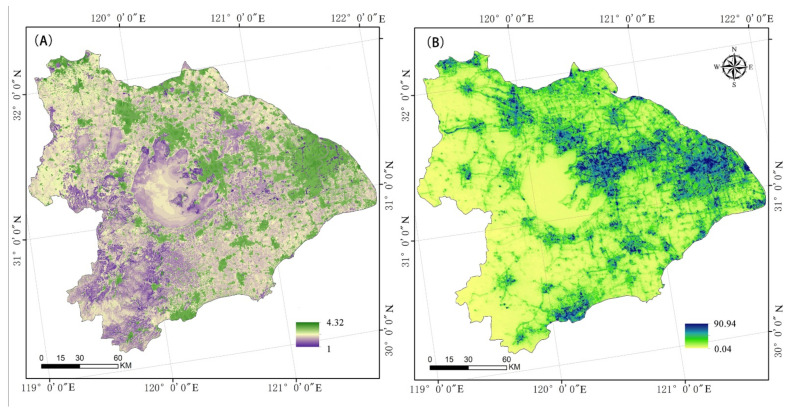
Resistance surface around Taihu Lake Basin: (**A**) Natural comprehensive resistance surface; and (**B**) corrected resistance surface.

Based on the height, slope, landscape type, vegetation covering, and distance from a river, a naturally integrated resistance surface for biodiversity was found to exist in the Taihu Lake basin (Figure 5A). Using information from night-time lighting, the drag surface of the Taihu Lake River basin was adjusted (Figure 5B). Shanghai, Suzhou, Wuxi, and Changzhou in Jiangsu Province, as well as Hangzhou and Jiaxing in Zhejiang Province, were found to be high-value areas. Although the Taihu Lake region was the most significant ecological supply for the Taihu Lake River basin, it also exhibited a high degree of ecological resistance. For this reason, we should focus on the areas around Taihu Lake and build an ecological network for the protection of biodiversity.

**Figure 6:**
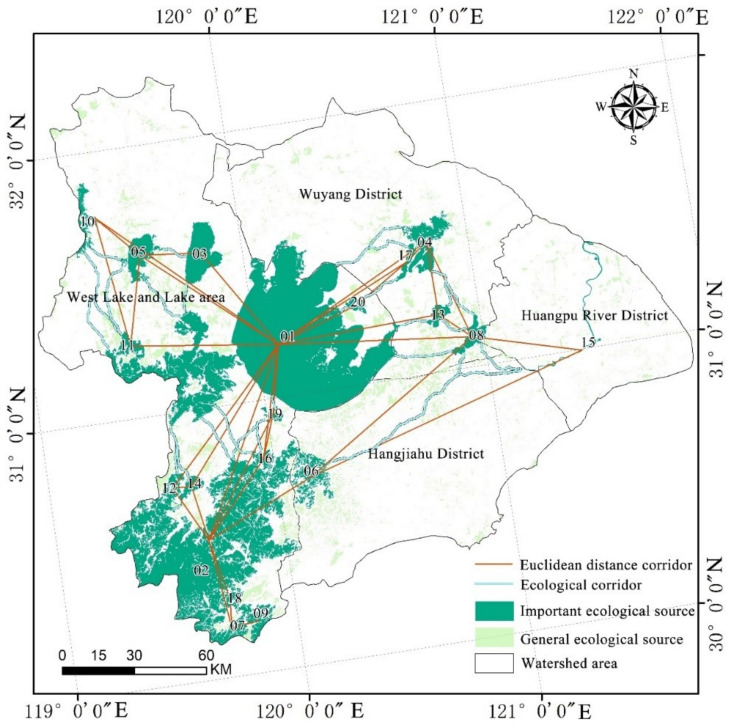
Ecological corridor distribution around Taihu Lake Basin.

Strips of biological land, known as corridors, should be made available to various species in order to connect ecosystems, avoid species isolation, maintain minimum numbers, and safeguard biodiversity. An ecological protection network is made up of ecological sources and corridors. In this line, 37 natural corridors with a combined length of 738.43 km were discovered (Figure 6). The corridors connected the habitats where species may thrive and could support the movement of organisms between the habitats. The absolute distance between sources in the study region is shown by red lines, which depict the Euclidean distance of the links between sources. The geometric center of gravity of each source served as the connecting element.

### 5.2. Evaluation of the ESP

Based on the global connectivity index, we calculated the significance of the core ecological sources suitable as various species habitats for the top 20 ecological sources. These 20 eco-source locations were also given site names, as listed in Table 5, making the results more useful and usable.

From the perspective of an ecological source of water, the lake body of Taihu Lake is the most important ecological source, providing an important channel for migratory birds. In the Suzhou area of Eastern Taihu Lake, the Yangcheng Lake Importance Score was 0.19, while the Chenghu index was 0.02. In contrast, the Huangpu River, which is primarily found in Shanghai, had a lower significance score (0.01), due to its limited width and great distance from other sources.

From the viewpoint of the ecological source of forest land, it was seen as an important living space for species, due to the large area of forest cover in the hills of western Zhejiang. The relevance index of the 02 forest source region, in comparison to the 01 Taihu Lake and mountains, was lower. According to the results, the relevance index of the source area and its area were not significantly positively correlated. For instance, Huzhou’s pictorial location No. 19 was just 30.83 km^2^ in size, yet its importance index reached 30.10. The above results indicate that we should pay more attention to how source regions are linked, and that the size of the ecological source is a factor affecting the survival of species.

In order to effectively guide the development and regulation of ecological networks in ecological regions, it is helpful to have a clear understanding of hot-spot locations and the directional aspects of ecological resistance impacting biodiversity. The entire Taihu Lake basin was partitioned into a 1 km × 1 km grid using ArcGIS, and this grid was utilized to create spatial statistics on the ecological resistance surface. The average grid resistance value is shown spatially, relative to the grid.

**Figure 7:**
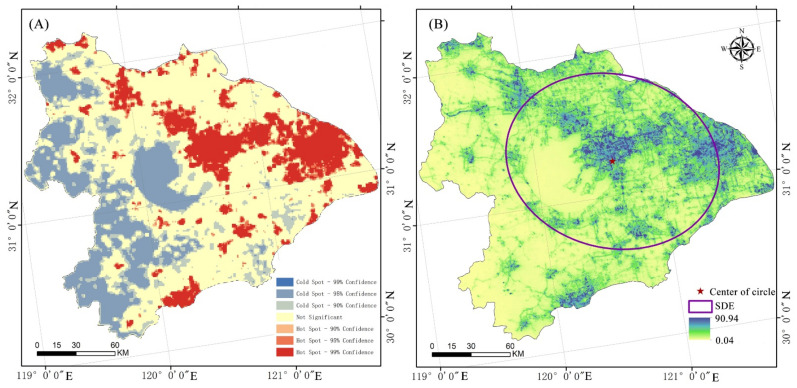
Characteristics of the ecological resistance in Taihu Lake Basin: (**A**) Cold and hot spot analysis; and (**B**) Standard deviation ellipse analysis.

As shown in Figure 7A, the spatial heterogeneity of habitat quality in the ecoregion around Taihu Lake was significant. Significant low-value areas included the lake body of Taihu Lake and the forested mountainous area in western Zhejiang Province. These areas are to the southwest of Taihu Lake. The ecological resistance hot-spot areas show a significant point–surface pattern distribution, where the centers of the clusters are mostly densely populated economic centers. The Wuxi-Suzhou-Shanghai line, on the other hand, displayed a continuous surface distribution, emphasizing the importance of cold- and hot-spots in space.

The standard deviation ellipse of the ecological resistance surface generally exhibited a northwest–southeast direction, as shown in Figure 7B, based on the study results of the ecological resistance direction for the ecological region around Taihu Lake. The azimuth angle was 104.13°, the long axis of the standard deviation ellipse was 66,611.03 km, and the short axis was 82,764.23 km. The center of gravity was situated east of the body of water in Taihu Lake. According to the continuous plane direction of the ecological resistance hot-spot, the general direction was to the east and north of Taihu Lake. The lake body of Taihu Lake and the forest mountain ecological source in Western Zhejiang were noteworthy, when combined with the findings of the ecological source study. The low-value biological source areas, on the other hand, were mostly located to the east of Taihu Lake, affected by both natural and man-made factors.

We established a computation using the ecological activity paths and various distances between different ecological sources, in order to fairly estimate the degree of interconnectedness between the ecological sources due to the significant gravitational differences between them (Table 6). Specifically, we covered the following five areas: (1) In the measuring space, the Euclidean distance represented the “normal” (i.e., straight line) separation between two ecological sources; (2) the cost-weighted distance can be thought of as an extension of the Euclidean distance, which assigns the ecological resistance cost factor to the distance passing through an ecological source; (3) the least expensive way between the two ecological sources, as measured by the unweighted length of the minimum cost path and the generated path, might be determined to be the best option in terms of the expense of constructing the ecological corridor; (4) the cost-weighted distance to Euclidean distance ratio (or Cwd/Euc) between ecological sources indicate that it was challenging for the straight-line distance between ecological sources to accurately reflect the actual value under diverse resistance situations; and (5) the ratio of the cost-weighted distance to the unweighted length of the minimum cost path (or Cwd/LCP) indicate how the channel connection between source locations affected how far the ecological path moved. 

### 5.3. Optimization of the ESP

Ecological nodes are the springboards and turning points for species in the ecological region, which are generally located at the weakest point, in terms of corridor function. They are primarily divided into key ecological protection nodes and key ecological restoration nodes, with the former being critical to ensuring that the ecological corridor effectively plays ecological functions and can guarantee the smoothness of the ecological corridor, while the latter is the area that hinders the confluence of the ecological corridor. In particular, consideration of the latter area, which hinders the connection between ecological sources, can allow us to greatly improve the landscape connectivity and ecological stability of the ecological region around Taihu Lake. For this reason, we measured and extracted 36 key ecological protection nodes and 24 key ecological restoration nodes.

**Figure 8:**
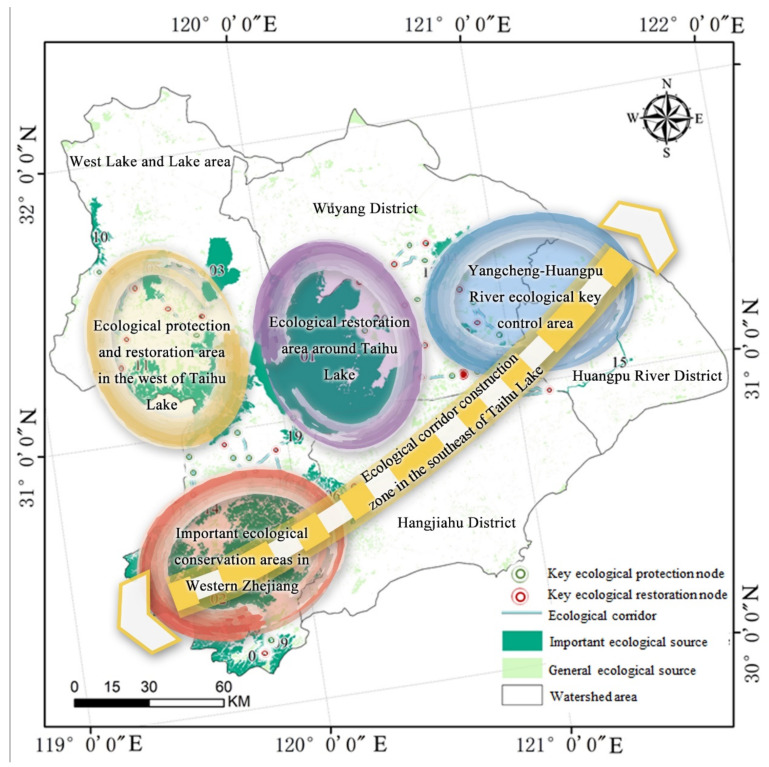
Optimization of the ESP around Taihu Lake Basin.

Furthermore, as most prior studies have only conducted quantitative analysis, we synthesized relevant planning policies issued by government units to improve the viability of ecological security strategies around the Taihu Lake basin, in light of emerging needs for national and regional strategic development. Basin plans were included, such as the Comprehensive Plan of the Taihu Lake Basin, the Comprehensive Plan of Flood Control for the Taihu Lake Basin, the Comprehensive Plan of Water Resources for the Taihu Lake Basin, and the Overall Plan for Comprehensive Management of the Water Environment around the Taihu Lake Basin. We further determined the spatial characteristics of the basin’s ecological network and joined all the basin’s parts to align the water resources to the national strategy and proposed a regulation strategy based on the ESP at the district and county scales for the Taihu Lake basin. Finally, as shown in Figure 8, we proposed the following “four zones and one belt” ecological optimization pattern: an ecological protection and restoration area in the west of Taihu Lake, an ecological restoration area around Taihu Lake, a Yangcheng-Huangpu River ecological key control area, an important ecological conservation area in Western Zhejiang, and an ecological corridor construction region to the southeast of Taihu Lake.

## 6. Discussion

### 6.1. Characteristics of the ESP

#### 6.1.1. Sources

Table 4 and Table 5 and Figure 4 above demonstrate the following: (1) The ranking of the various ecological source categories was as follows: core, edge, branch, pore, bridge, deserted island, and ring. It is challenging for ecological sources with a fragmented layout to exert complete economic and ecological benefits, as they make up a relatively small fraction of the types of ecological sources with circulation functions, isolating them from one another [54]. The hilly southwest and Taihu Lake were the main geographic areas, comprising 9145.86 km^2^, or 85.23% of the total area. There is a clear need for increased research into cross-administrative ecological and environmental protection governance, as well as unified planning and administration in the region. (2) The first 20 core ecological sources were mainly water and forest/grassland, among which the water ecological sources were mainly the scattered wetland landscape areas around the Taihu Lake basin, and the forest/grassland ecological sources were concentrated in the hilly and mountainous areas in the southwestern part of the Taihu Lake eco-region. The main goals of regional sustainable development in the study area should involve the protection of water areas and hilly forest lands. The above-mentioned findings are in agreement with earlier research [7]. (3) The connectivity between ecological sources will determine the theoretical foundations for further research on ecological corridors and important nodes and will have an effect on the importance index.

#### 6.1.2. Corridors

Table 6 and Figure 5, Figure 6 and Figure 7 above demonstrate the following: (1) The ecological resistance surface will affect the effectiveness of the ecological corridor [55]. The Taihu Lake Basin’s rectified resistance surface analysis indicate significant spatial variation in the research region. A pattern of “one center and two axes” is shown in the high-value area. The Suzhou Xichang town group and the Qiantang River town group form the axes, while Shanghai is the center. It is evident that the creation of biological corridors around Taihu Lake was significantly impacted by urbanization. (2) The Taihu Lake Basin region contains 37 ecological corridors, most of which connect the area’s extensive river network and forested areas. Given the contradiction between environmental protection and economic growth, and the fact that these various rivers and mountains serve as corridors for species migration [56,57], the corridors must be connected to the source edge. (3) The ecological source and corridor comprise a regional ecological protection network. The Taihu Lake area’s crucial ecological corridor stretches eastward to Chenghu Lake. It crosses Taihu Lake and connects the mountainous forest in Western Zhejiang with the important lakes in the region. The lowest Cwd/LCP ratios were found in the source areas of Taihu Lake and Mountain, Tianmu Mountain, Gehu Lake, Changing Lake, Maoshan Scenic Area, and Zicheng Scenic Area of Huzhou City. These ecological sources are highly significant. Therefore, it is important to ensure the preservation of lakes and the area between lakes and forest land.

#### 6.1.3. Nodes

**Figure 9:**
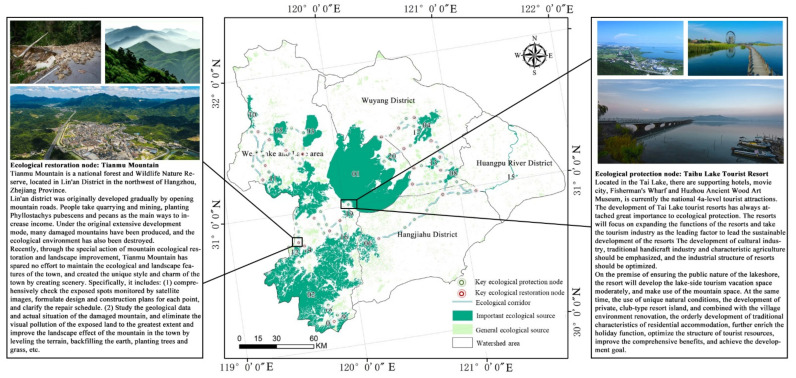
Two key nodes of ecological protection and restoration around the Taihu Lake Basin.

Table 4 and Table 5 and Figure 8 above demonstrate that: (1) The 24 crucial ecological restoration nodes and the 36 crucial ecological preservation nodes were primarily extracted along movement corridors (sometimes called ecological corridors) or ecological paths [58]. Therefore, for case analysis, typical key nodes should be chosen. Alternative tactics were also suggested (Figure 9). Among them, the Suzhou Taihu National Eco-Resort Area is a typical ecological protection node with the ecological environment of Taihu Lake as its resource base, responding to the needs of social development and integrating the rich ecological resources of the region with the development of an ecological service industry as the main line. In Hangzhou, Zhejiang Province, the original intensive development pattern of Tianmu Mountain resulted in the destruction of numerous mountains as well as the ecological environment. Tianmu Mountain is attempting to strike a balance between ecological restoration and economic development, in accordance with the specific action plan for the ecological restoration and landscape improvement of the mountains “welcoming the Asian Games” in Lin’an District, Hangzhou City. By modifying and breaking down ecological restoration tasks, creating a treatment plan, and condensing the primary duty subjects, specific action may be continuously encouraged. This node is typical of ecological restoration.

In conclusion, the upgrade needs of ecological restoration nodes are the most pressing. The effect will also be the most obvious, once the measures are greatly improved. The overall efficiency of connections can be directly enhanced by ecological restoration in this area. Ecological protection nodes are dispersed extensively in the area, and improvement in the area is taking place gradually. As a result, it comprises work that requires ongoing maintenance and improvement.

### 6.2. Optimization Path for the ESP in Ecological Regions

#### 6.2.1. Partition Strategy

In this study, we propose the zoning plan of “four zones and one belt” for ecological security surrounding Taihu Lake, based on the premise of “preservation first and development at the same time.” The key details are as follows:

(1) An important ecological conservation area in Western Zhejiang—This is a significant natural conservation region. With low ecological resistance and great habitat quality, this region is a crucial ecological source for the Taihu Lake Basin. Therefore, it should be against the law for humans to disrupt the natural ecology and for any kind of development that is not tied to protecting the environment to have an effect on the living environment.
(2) An ecological protection and restoration area in the west of Taihu Lake: Huzhou, Wuxi, and Changzhou—These ecological settings are favorable and provide better living conditions for species. On the other hand, Changzhou and other locations have experienced significant economic growth and are situated at the intersection of ecological resistance and cold and hot zones of habitat quality. Human interference factors are expected to have a significant impact on biological activity. Therefore, the site should be reasonably designated as an ecological “red line” for protection and restoration of the damaged ecological environment. The main goal is to keep people from moving into biologically important nodes and ecological corridors due to rapid economic growth.
(3) An ecological restoration area around Taihu Lake—Wuxi, Suzhou, and Huzhou are in this region, among other cities. The surrounding area of Taihu Lake is also a region with high ecological resistance, which limits the migration routes of land animals to Taihu Lake. The body of Taihu Lake is the most important ecological source in the Taihu Lake Basin. As a result, it is critical to increase the promotion of water ecological restoration projects with a focus on wetland reconstruction, aquatic vegetation restoration, and the construction of ecological protection forests around Taihu Lake. Additionally, the ecological land corridor from Taihu Lake to each source must be opened.
(4) The Yangcheng-Huangpu River ecological key control area: Jiaxing, Suzhou, and Shanghai—There are just four biological source regions in this area, and Taihu Lake is not sufficiently connected. Low habitat quality and most of them being situated in hot-spots and circular zones of ecological resistance have a significant impact on the function of ecological sources. Therefore, the site requires improved management and control of the ecological environment. The river corridor should also be widened, along with the development of more buffer zones to protect the environment on both sides of the Taipu River, which runs between Taihu Lake and Dianshan Lake.
(5)  An ecological corridor construction zone in the southeast of Taihu Lake: Shanghai, Jiaxing, Huzhou, and other locations—There is currently no ecological connection between the highland source areas in Western Zhejiang and ecological sources such as Dianshan Lake. The planned area for the corridor contains a small percentage of natural area, and the function of biodiversity protection is unclear. To encourage the East–West linkage of the sources, it is necessary to appropriately minimize land construction, enhance lake and river network environmental governance, and implement ecological restoration. Additionally, a shoreline ecological buffer zone should be developed.

#### 6.2.2. Protection and Utilization

First, we followed the principle of “protection first.” The primary premise of the ecological region around Taihu Lake is to give priority to protection. To protect important ecological sites such as mountains, rivers, and lakes—as well as important sources, corridors, and nodes—all administrative units in the region should follow the principles of integrity, unified planning, unified monitoring, and unified action.

Secondly, the Taihu Lake basin is difficult to unify and coordinate, as it spans multiple administrative regions, and the continuous development of urbanization will inevitably lead to a coercive effect on the ecological environment [7,59]. One of the key points that urgently needs to be addressed is how to coordinate the many contradictions across the multiple administrative regions of the basin. Therefore, first we should establish and improve the joint prevention and control mechanism of the ecological environment around Taihu Lake basin, establish a unified coordination and dialogue platform, and clarify the rights of each location through relevant laws. Next, the creation of a compensation mechanism for protecting the environment around the Taihu Lake basin should be prioritized, the value of ecological products around the Taihu Lake basin should be increased, and talks should be conducted with other countries, in order to learn how to develop Taihu Lake in a way that is beneficial for everyone.

Third, as China enters an era of mass leisure, ecological regions have become the main bearing space for leisure and recreation [60], and the integrated development of ecology and recreation is conducive to enhancing the compound value of ecological regions and promoting the well-being of local residents [61]. For this reason, such spaces must be protected, such that green businesses (e.g., ecotourism and leisure agriculture) can grow.

### 6.3. Limitations and Prospects

The current study had some shortcomings. First, although the night-time lighting index-modified ecological resistance coefficient was included in the construction of the ecological resistance surface, there are still some socio-economic factors that were not included [62], and future studies should fully consider the influence of natural and social factors. Second, the ESP can change significantly over time, and studying its trends can provide an important reference for ecological restoration or conservation [6]. Therefore, in the future, we will progress to studying the temporal trends of the ESP in the Taihu Lake region. Third, the combination of qualitative and quantitative methods is an expected future trend of the ESP research [5]. In this line, we plan to use field surveys and interview questionnaires to conduct field research on ecologically important places and businesses near Taihu Lake. This will help us learn more about the best way to use the ESP in the ecological region around Taihu Lake.

## 7. Conclusions

In this paper, we combined the MSPA-MCR model, took the ecological region around Taihu Lake as a typical study area, focused on the spatial distribution characteristics of ecological elements in the study area from the perspective of biological habitats, and quantitatively analyzed the spatial distribution characteristics of ecological elements in the study area. Based on this, a regulatory framework for the ESP in the ecological region around Taihu Lake was proposed. The conclusions of the study are as follows:

(1) Sources: Based on the MSPA, the top 20 ecological sources were identified and ranked, according to the dPC importance index. These included important lakes and forests, among which Lake Taihu was the most important ecological source. Most of the sources were in the western part of Zhejiang and the area west of Taihu Lake, forming a clear dividing line.
(2) Corridors: We determined the effect of connectivity between ecological sources based on relevant distance measurements. The ecological corridors ran through Lake Taihu and its mountains to connect the sources to each other, mainly in areas with less ecological resistance.
(3) Nodes: Based on a plug-in in the Linkage Mapper toolbox, 36 key ecological protection nodes and 24 key ecological restoration nodes in the study area were extracted. These may be considered as priority areas for future improvement.
(4) Spatial characteristics of ecological resistance: The hot-spots of ecological resistance were concentrated in the northeastern part of Taihu Lake, and the standard deviation ellipse also deviated to the northeast. Meanwhile, most of the cold-spots were along rivers and lakes with healthy natural ecosystems, as well as in the woods around them.
(5) Optimization path: Based on the spatial characteristics of the Taihu Lake basin and planning and development needs, the ESP was developed based on a “four zones and one belt” pattern. At the spatial level, a regulation strategy based at the district and county scales was suggested. At the governance level, principles of protection and utilization were suggested.

Finally, the study’s shortcomings were examined, and suggestions for future research directions were made.

## Figures and Tables

**Table 1:** Data sources.

Data Type	Source	Explain
Regional boundary around Taihu Lake	Resources and environment science and data center of Chinese Academy of Sciences (https://www.resdc.cn/, accessed on 15 June 2022).	The four river basins of Huxi and Hu-zhou District, Wuyang District, and Huangpu River District are formed by the geographical distribution of China’s three-level river basins.
River water area around Taihu Lake	National Geographic Information Resources Directory Service System (https://www.webmap.cn/commres.do?method=result100W, accessed on 1 June 2022).	The Ministry of Natural Resources has granted permission to provide the service of free downloading of water layer materials.
Land-use data in 2020	Global land-cover data set globeland30 (http://www.globallandcover.com/, accessed on 12 June 2022).	Extract land-use information with a 30 m spatial resolution from the research region.
DEM data	ASTERGDEM digital elevation data derived from Chinese Academy of Sciences Geospatial Data Cloud with 30 m resolution (http://www.gscloud.cn/, accessed on 3 June 2022).	The slope will also be extracted from these values at the same time.
Normalized Difference Vegetation Index (NDVI)	From the Chinese Academy of Sciences Geospatial Data Cloud (https://www.gscloud.cn/, accessed on 8 June 2022).	In particular, the 250 m-resolution MOD13Q1 products are used.
Night-time light data	Using 2020 NPP-VIIRS Data (https://www.ngdc.noaa.gov/, accessed on 10 June 2022).	The scientific validity of using night-time illumination data to reflect factors such as regional urbanization, socio-economic status, environmental energy consumption, and so on was previously established [32]. Before putting all the 2020 data together, it was processed and fixed with the help of an algorithm.

**Table 2:** Landscape types and ecological significance of MSPA.

Landscape Type	Ecological Significance
Core	Significant for biodiversity protection and can be viewed as the “source” of the ecological process as it is a crucial habitat patch in terms of landscape features.
Bridge	Ecological corridors are the long, slender stretches that connect patches in various core areas. They are mostly green belts that look like belts and help species move around and connect.
Edge	A transition zone between the edge of the core region and the non-green landscape area on the periphery can reduce the effects of external factors and human disturbances.
Loop	Putting the inner channels together can speed up the exchange of matter and energy in the same core area.
Perforation	Between the core area and the non-green ecological patch, there is a transition zone that is thought to have an edge effect.
Branch	This area is the main extension of the green belt. It has only one end that connects to the main patch but acts as a way for species to move around and energy to flow between the green belt and the land around it.
Islet	The interchange of matter and energy between disconnected, dispersed patches is exceedingly rare. Most of these patches are modest green areas in urban or rural settings.

**Table 3:** Resistance factors and associated values.

Evaluation Factor	Grading Indicators	Resistance Value	Weight
Types of land-use	Forest and grassland	1	0.480
	Water area (including wetland)	2	
	Cultivated land	3	
	Naked	4	
	Land used for building	5	
Slope	<5°	1	0.173
	5°–15°	2	
	15°–25°	3	
	25°–35°	4	
	>35°	5	
DEM (Digital Elevation Data)	<50 m	1	0.152
	50–150 m	2	
	150–250 m	3	
	250–500 m	4	
	>500 m	5	
NDVI	>0.65	1	0.090
	0.5–0.65	2	
	0.35–0.5	3	
	0.15–0.35	4	
	<0.15	5	
Distance from water	Natural fracture method	1–5	0.105

**Table 4:** Statistical results of the MSPA classification.

Landscape Type	Total Area (km^2^)	Proportion in the Outlook	Proportion in the Study Area
Core	9145.86	85.23%	25.03%
Islet	41.18	0.38%	0.11%
Perf	163.88	1.53%	0.45%
Edge	1071.49	9.99%	2.93%
Loop	25.77	0.24%	0.07%
Bridge	92.63	0.86%	0.25%
Branch	189.56	1.77%	0.52%

**Table 5:** Ecological source importance index results.

Number	Area (km^2^)	Name of Place	dPC Index
01	3339.14	Taihu Lake and mountain	73.71
02	2125.00	Tianmu Mountain	54.40
03	244.03	Gehu Lake	0.36
04	191.52	Yangcheng Lake	0.19
05	146.75	Changdang Lake	0.22
06	136.90	Xiazhu Lake Wetland Scenic Area	4.01
07	89.71	Around Tianmu Mountain	3.32
08	78.48	Dianshan Lake	0.04
09	74.75	Around Tianmu Mountain	2.25
10	69.83	Maoshan Scenic Area	0.02
11	57.63	Yunhu Scenic Area	1.95
12	55.46	Around Tianmu Mountain	1.77
13	47.75	Chenghu Lake	0.02
14	46.29	Xialin Jiutian silver Waterfall Scenic Area	1.39
15	39.45	The Huangpu River	0.01
16	39.15	Mogan Mountain Scenic Area	1.20
17	36.05	Yangcheng West Lake	0.06
18	33.16	Around Tianmu Mountain	1.02
19	30.83	Zicheng scenic area of Huzhou City	30.10
20	30.61	Dayangshan National Forest Park	0.85

**Table 6:** Ecological corridor path and distance calculation.

Number	Ecological Path	Euclidean Distance (Euc)/km	Cost-Weighted Distance (Cwd)/km	Unweighted Length of Minimum Cost Path (LCP)/km	Cost-Weighted Distance/Euclidean Distance (Cwd/Euc)	Cost-Weighted Distance/Unweighted Length of Minimum Cost Path (Cwd/LCP)
1	10-05	11.77	2.97	12.84	0.25	0.23
2	10-11	35.57	13.24	42.43	0.37	0.31
3	10-01	39.53	13.12	47.99	0.33	0.27
4	04-17	0.03	1.01	0.60	33.68	1.70
5	04-13	13.04	53.95	20.70	4.14	2.61
6	04-08	25.24	65.84	29.50	2.61	2.23
7	04-01	21.22	60.55	36.27	2.85	1.67
8	07-20	13.71	85.78	15.33	6.26	5.60
9	17-01	16.33	69.75	37.82	4.27	1.84
10	03-05	9.30	6.09	11.37	0.65	0.54
11	05-11	19.87	12.70	34.30	0.64	0.37
12	05-01	20.03	11.87	23.68	0.59	0.50
13	03-01	10.47	8.57	13.55	0.82	0.63
14	20-01	3.09	9.83	3.49	3.18	2.81
15	13-08	9.03	11.34	12.40	1.26	0.92
16	13-01	11.12	24.56	12.19	2.21	2.01
17	08-15	19.21	15.86	24.89	0.83	0.64
18	08-01	23.35	38.70	25.67	1.66	1.51
19	08-06	68.37	66.83	83.91	0.98	0.80
20	11-01	0.18	0.04	0.29	0.23	0.14
21	06-15	88.20	77.97	103.11	0.88	0.76
22	19-01	2.88	2.58	5.71	0.90	0.45
23	19-16	11.39	6.36	16.03	0.56	0.40
24	19-02	9.06	7.69	23.47	0.85	0.33
25	01-16	19.24	7.60	28.27	0.40	0.27
26	01-12	19.14	5.02	20.87	0.26	0.24
27	01-14	21.65	6.06	24.37	0.28	0.25
28	01-02	11.89	5.43	22.47	0.46	0.24
29	02-16	0.07	0.03	0.12	0.43	0.24
30	12-14	0.27	0.10	0.32	0.37	0.31
31	02-12	0.06	0.02	0.13	0.38	0.17
32	02-14	1.24	1.03	3.08	0.83	0.33
33	02-06	0.53	0.22	0.58	0.42	0.38
34	02-18	0.03	0.06	0.07	2.24	0.90
35	18-07	0.23	0.97	0.29	4.24	3.35
36	02-07	0.17	0.06	0.21	0.38	0.30
37	07-09	0.07	0.12	0.11	1.74	1.02

Note: The numbers in the ecological paths are consistent with those in Table 5.

## Data Availability

The original contributions presented in the study are included in the article; further inquiries can be directed to the corresponding author.
